# The HD-Zip II Transcription Factor *SlHZ07* Promotes Growth and Drought Tolerance in Tomato

**DOI:** 10.3390/antiox15091062

**Published:** 2026-08-25

**Authors:** Shuchao Dong, Jiaxin Li, Jingwen Zhang, Liuxia Song, Yinlei Wang, Liping Zhao, Jie Chen, Yariv Brotman, Junming Li, Tongmin Zhao

**Affiliations:** 1 Jiangsu Key Laboratory for Genetic Improvement and Efficient Utilization of Horticultural Crops, Institute of Vegetable Crops, Jiangsu Academy of Agricultural Sciences, Nanjing 210014, China; 2 State Key Laboratory of Vegetable Biobreeding, Institute of Vegetables and Flowers, Chinese Academy of Agricultural Sciences, Beijing 100081, China; 3 College of Horticulture and Plant Protection, Yangzhou University, Yangzhou 225100, China; 4 College of Horticulture, Nanjing Agricultural University, Nanjing 210095, China; 5 School of Plant Sciences and Food Security, Institute for Cereal Crops Research, Tel Aviv University, Tel Aviv 6997801, Israel

**Keywords:** tomato, HD-Zip TF, *SlHZ07*, drought tolerance, ROS, JA, GA

## Abstract

Drought is one of the major environmental constraints limiting tomato growth and productivity. Identifying regulators that enhance drought tolerance without compromising plant growth is therefore important for tomato production. Homeodomain-leucine zipper (HD-Zip) transcription factors (TFs) play essential roles in plant development and abiotic stress responses; however, the functions of most HD-Zip II members in tomato remain poorly understood. Here, we identified *SlHZ07*, a drought-responsive HD-Zip II TF, through transcriptome analysis and characterized its biological function in tomato. *SlHZ07* was rapidly induced by drought stress and localized predominantly to the nucleus. Overexpression of *SlHZ07* significantly enhanced drought tolerance, whereas RNAi-mediated suppression increased drought sensitivity. Physiological analyses showed that *SlHZ07* overexpression reduced reactive oxygen species (ROS) accumulation, enhanced antioxidant enzyme activities, upregulated expression of ROS-scavenging genes, and alleviated membrane damage under drought stress. Hormone analyses revealed that *SlHZ07* positively regulated jasmonic acid (JA) accumulation and the expression of JA biosynthetic genes, including *OPR2*, *OPR3*, *JAR1*, and *AOC*, but did not alter endogenous abscisic acid (ABA) levels under well-watered conditions. Furthermore, *SlHZ07* promoted vegetative growth by increasing endogenous gibberellin (GA) levels and upregulating the expression of the GA biosynthetic genes *GA20ox2* and *GA20ox4*. Together, our findings identify *SlHZ07* as a previously uncharacterized positive regulator that coordinates plant growth and drought adaptation by integrating GA biosynthesis, JA homeostasis, and ROS detoxification. These results expand our understanding of HD-Zip II TFs and provide a promising genetic target for improving drought tolerance in tomato.

## 1. Introduction

Tomato (*Solanum lycopersicum*) is one of the most extensively cultivated and economically important vegetable crops worldwide, ranking first among vegetable species in both cultivation area and total production [1,2]. However, its productivity is highly vulnerable to environmental stimuli, among which drought is one of the most detrimental under current climate change scenarios [3]. Owing to its relatively high-water demand, particularly during reproductive development, tomato is highly sensitive to water deficit, which frequently results in reductions in vegetative growth, fruit quality, and fruit yield [4,5]. At the physiological level, drought stress impairs photosynthesis, nutrient acquisition, cellular homeostasis, and reproductive development, ultimately limiting productivity [6,7]. As the frequency and severity of drought events continue to increase worldwide, enhancing drought tolerance while minimizing growth penalties has become a major objective of tomato breeding and molecular research [8]. A major challenge is to understand how tomato plants coordinate growth-promoting processes with drought-adaptive responses, which may otherwise compete for limited resources under drought conditions.

Drought adaptation is governed by complex regulatory networks involving stress perception, phytohormone signaling, ROS homeostasis, osmotic adjustment, and extensive transcriptional reprogramming [9,10]. TFs are key regulators of these regulatory networks, which coordinate the expression of numerous downstream genes in response to environmental stimuli [11]. Several TF families, including AP2/ERF, NAC, bZIP, MYB, and bHLH, have been shown to enhance drought tolerance by regulating ABA signaling, antioxidant defense, osmoprotectant biosynthesis, and stomatal movement [9,10,12,13,14]. Nevertheless, the transcriptional networks underlying drought adaptation in tomato remain incompletely characterized, and the functions of many stress-responsive TFs have yet to be determined.

The plant-specific HD-Zip TF family is characterized by a highly conserved homeodomain (HD) that mediates DNA binding and an adjacent leucine zipper (Zip) motif required for homo- or heterodimerization [15]. The four HD-Zip subfamilies (HD-Zip I-IV) have diverse developmental and stress-related functions [16]. Members of the HD-Zip I and II subfamilies are particularly responsive to abiotic stresses and phytohormone signaling, whereas HD-Zip III and IV proteins primarily regulate meristem maintenance, organ polarity, vascular development, and epidermal differentiation [17,18,19]. For instance, the HD-Zip I TF AtHB13 positively regulates drought tolerance by activating the NAC TF *AtNAC042* in Arabidopsis [20]. Likewise, the maize HD-Zip I TF ZmHDZ9 confers drought resistance by modulating ABA signaling and lignin accumulation [21]. These findings suggest that HD-Zip proteins may serve as important regulators linking plant development with environmental adaptation. However, the functional characterization of HD-Zip proteins in tomato remains limited.

A previous genome-wide analysis identified 51 HD-Zip genes (*SlHZ01*–*SlHZ51*) in the tomato genome, many of which are responsive to environmental stresses, including cold, salinity and dehydration stress [22,23,24]. Nevertheless, only a limited number of tomato HD-Zip TFs have been functionally characterized. For example, silencing the HD-Zip III gene *SlHDZ34* compromised salt tolerance [24], whereas the HD-Zip I TF SlHZ24 enhanced oxidative stress tolerance by promoting ascorbate biosynthesis [25]. Importantly, whether tomato HD-Zip proteins can coordinate plant growth with drought adaptation remains largely unknown. We previously identified *SlHZ07* (*Solyc02g063520*), an HD-Zip II TF, as a dehydration-responsive gene in tomato leaves [23]. However, its biological function and the molecular mechanisms by which it may contribute to drought adaptation have not been determined. In particular, whether *SlHZ07* acts as a regulatory link between growth and stress responses represents an important unanswered question.

In this study, we investigated the biological function and regulatory mechanisms of *SlHZ07* in tomato growth and drought tolerance. We demonstrate that *SlHZ07* acts as a positive regulator of both vegetative growth and drought tolerance in tomato. Hormone profiling combined with gene expression analyses further revealed that *SlHZ07* modulates GA and JA homeostasis by promoting the expression of key biosynthetic genes. Furthermore, physiological and biochemical analyses showed that *SlHZ07* enhances drought tolerance by reducing ROS production, strengthening antioxidant capacity, and preserving membrane integrity under water-deficit conditions. Collectively, these findings identify *SlHZ07* as a regulator connecting growth-related and drought-responsive processes, providing insights into HD-Zip II TFs-mediated stress regulation and a promising genetic target for improving drought tolerance in tomato.

## 2. Materials and Methods

### 2.1. Plant Material and Growth Conditions

The *S. lycopersicum* cv. MicroTom (MT) was used as wild-type plant materials in this study. Seeds were initially germinated on full-strength Murashige and Skoog medium supplemented with 2% (*w*/*v*) sucrose. After two weeks of growth, seedlings were transplanted individually into pots (9 cm diameter) containing a 2:1 (*v*/*v*) mixture of potting soil and quartz sand. Plants were maintained in a controlled-environment chamber at 25 °C under a 14 h light/10 h dark photoperiod provided by fluorescent lamps, with a relative humidity of approximately 75% and a photosynthetic photon flux density of 500 µmol m^−2^ s^−1^. Plants were supplied with nutrient solution once per week. For drought stress assays, plants were grown under well-watered conditions for six weeks after germination. Each plant was cultivated in approximately 100 ± 5 g of soil, and drought stress was subsequently induced by completely withholding irrigation for 0–9 days. Fifteen plants from each genotype were used in each treatment.

### 2.2. Plasmid Construction and Plant Transformation

For the *35S-SlHZ07-GFP* construct, the full-length coding sequence (CDS) of *SlHZ07* without stop codon was PCR-amplified and subsequently introduced into the pENTR/D-TOPO entry vector using the pENTR™ Directional TOPO^®^ Cloning Kit (Invitrogen, Carlsbad, CA, USA). Following sequence verification by Sanger sequencing (Sangon Biotech, Shanghai, China), the insert was recombined into the destination vector pK7FWG2 [26] by LR recombination (Invitrogen). For RNA interference (RNAi) construct generation, the *SlHZ07* RNAi target fragment was cloned into the vector pK7GWIWG2 [26] in both sense and antisense orientations by Gateway cloning (Invitrogen). All constructs were introduced into *Agrobacterium tumefaciens* strain GV2260 and subsequently transformed into MT tomato by *Agrobacterium*-mediated transformation. Primer sequences used for vector construction are provided in Table S1.

### 2.3. Quantitative Real-Time PCR (qRT-PCR)

Total RNA was extracted and first-strand cDNA was synthesized as described previously [27]. For RNA isolation, leaf tissue was homogenized to a fine powder in liquid nitrogen. Total RNA was then extracted using the RNAprep Pure Plant Plus Kit (TIANGEN, Beijing, China) following the manufacturer’s protocol. First-strand cDNA was prepared from 1 μg of purified RNA using the FastKing cDNA First-Strand Synthesis Kit (TIANGEN, Beijing, China). Gene-specific primers were designed using QuantPrime [28] and synthesized by Tsingke (Beijing, China). qRT-PCR was performed using a QuantStudio™ 6 Flex Real-Time PCR System (Applied Biosystems, Foster City, CA, USA) with SYBR Green chemistry (Vazyme, Nanjing, China). The amplification program consisted of an initial denaturation at 95 °C for 30 s, followed by 40 cycles of 95 °C for 10 s and 60 °C for 30 s. Relative transcript levels were normalized to reference gene *SlACTIN* (*Solyc11g005330*), which was previously used as a reference gene in tomato under abiotic stress conditions [7,24]. Primer sequences used for qRT-PCR are provided in Table S2.

### 2.4. Phytohormone Analysis

Frozen leaf samples were ground twice in liquid nitrogen (50 Hz, 30 s). A 100 mg aliquot was extracted with 1 mL precooled (−40 °C) acetonitrile: methanol (1:1, *v*/*v*) containing internal standards, vortexed (60 s), sonicated (10 min, ice water), incubated (2 h, −40 °C), and centrifuged (15 min, 12,000 rpm, 4 °C). The supernatant (900 μL) was dried, reconstituted in 90 μL 50% methanol, vortexed, sonicated, and centrifuged twice (10 min, 12,000 rpm, 4 °C). A 70 μL aliquot of the clarified extract was transferred to auto sampler vials for ultra-high-performance liquid chromatography-MS/MS (UHPLC-MS/MS) at Genepioneer, Nanjing, China (http://www.genepioneer.com/). Metabolite concentration (nmol/g) was calculated by the calibration curve.

### 2.5. Confocal Laser Scanning Microscopy

To visualize nuclear localization, tomato leaf cells were incubated with 10 mg/L 4′,6-diamidino-2-phenylindole (DAPI) solution for 30 min to stain the nuclei. *SlHZ07*-GFP fluorescence was subsequently examined by confocal laser scanning microscopy (CLSM) following the procedure described previously [29].

### 2.6. RNA-Seq Analysis

RNA-seq library construction and sequencing were performed at Genepioneer, Nanjing, China (http://www.genepioneer.com/). Three independent biological replicates were analyzed for each sample, and sequencing was carried out on the Nova 6000 platform (Illumina, San Diego, CA, USA). Raw sequencing data were processed with Trimmomatic to remove adapter sequences and reads of low quality [30]. The resulting clean reads were mapped to the tomato SL4.0 reference genome using HISAT2-3N [31]. Gene expression levels were quantified as fragments per kilobase of transcript per million (FPKM) using StringTie, with calculations based on gene length and the number of reads assigned to each gene [32]. Differentially expressed genes (DEGs) were identified using the DESeq R package, applying an adjusted *p*-value cutoff < 0.05 together with an absolute fold change ≥ 2 as the significance criteria [33]. The raw RNA-seq datasets have been deposited in the CNCB BioProject database (https://ngdc.cncb.ac.cn/bioproject/, accessed on 10 June 2026) under accession number PRJCA063061.

### 2.7. Determination of Relative Water Content (RWC) and Ion Leakage

The RWC of leaves was assessed according to a previously reported method [9]. Briefly, freshly excised leaves were weighed immediately to obtain the fresh weight (FW), followed by immersion in distilled water at 4 °C overnight to allow complete rehydration. The leaves were then weighed to determine the saturated weight (SW) and subsequently dried at 60 °C for 24 h to obtain the dry weight (DW). RWC was calculated as follows: RWC% = (FW − DW)/(SW − DW) × 100%. Membrane integrity was evaluated by measuring electrolyte leakage. Detached tomato leaves were placed in 50 mL of deionized water and gently shaken at room temperature for 12 h. The initial electrical conductivity was recorded at room temperature using a conductivity meter (INESA, Shanghai, China). The samples were then boiled at 100 °C for 30 min and cooled to room temperature before the final conductivity was determined. Electrolyte leakage was expressed as the ratio of the initial conductivity to the final conductivity after boiling.

### 2.8. Enzyme Measurements

Antioxidant enzyme activities were determined following previously described procedures [23]. Samples from three biological replicates were collected, frozen in liquid nitrogen, and ground to a fine powder. A 100 mg aliquot of each sample was homogenized in 500 µL lysis buffer containing 0.1 mM EDTA, 50 mM Tris–HCl (pH 7.8), 1% (*w*/*v*) polyvinylpolypyrrolidone (PVPP), and 0.1% (*w*/*v*) Triton X-100. After centrifugation at 10,000× *g* for 10 min, the resulting supernatants were collected for enzyme activity assays. Commercial kits for peroxidase (POD, EC 1.11.1.1), catalase (CAT, EC 1.11.1.6), superoxide dismutase (SOD, EC 1.15.1.1), and ascorbate peroxidase (APX, EC 1.11.1.11) (JC DETECT, Nanjing, China) were employed according to the manufacturer’s instructions. Absorbance was recorded using an Infinite 200 Pro M Nano plate reader (TECAN, Männedorf, Switzerland). Each measurement was performed in technical duplicate.

### 2.9. Quantification of Malondialdehyde (MDA), Hydrogen Peroxide (H_2_O_2_) and Superoxide Anion (O_2_•^−^)

The levels of H_2_O_2_ and O_2_•^−^ were determined using commercial assay kits (JC DETECT, Nanjing, China). Lipid peroxidation was evaluated by quantifying MDA with a corresponding assay kit from the same supplier. All measurements were conducted according to the manufacturers’ protocols, and absorbance was recorded using an Infinite 200 Pro M Nano plate reader (TECAN, Männedorf, Switzerland).

### 2.10. Yeast One-Hybrid (Y1H) Analysis

A 550 base pair (bp) fragment of the *JAR1* promoter containing the predicted HD-Zip binding site was amplified and cloned into the pAbAi vector to generate the bait construct *pAbAi-JAR1pro*, which was subsequently integrated into the yeast strain Y1HGold. The CDS of *SlHZ07* was cloned into the pGADT7 vector to generate the prey construct *pGADT7-SlHZ07-AD*, which was then transformed into the Y1HGold strain carrying *pAbAi-JAR1pro*. The interaction between *SlHZ07* and the 550 bp *JAR1* promoter fragment were evaluated on SD/-Leu medium supplemented with or without 50 ng/mL aureobasidin A (AbA). Growth on selective medium containing AbA was used to assess the binding of *SlHZ07* to the *JAR1* promoter and to eliminate false-positive interactions.

## 3. Results

### 3.1. *SlHZ07* Is a Drought-Responsive Gene Identified by Transcriptomic Analysis

**Figure 1:**
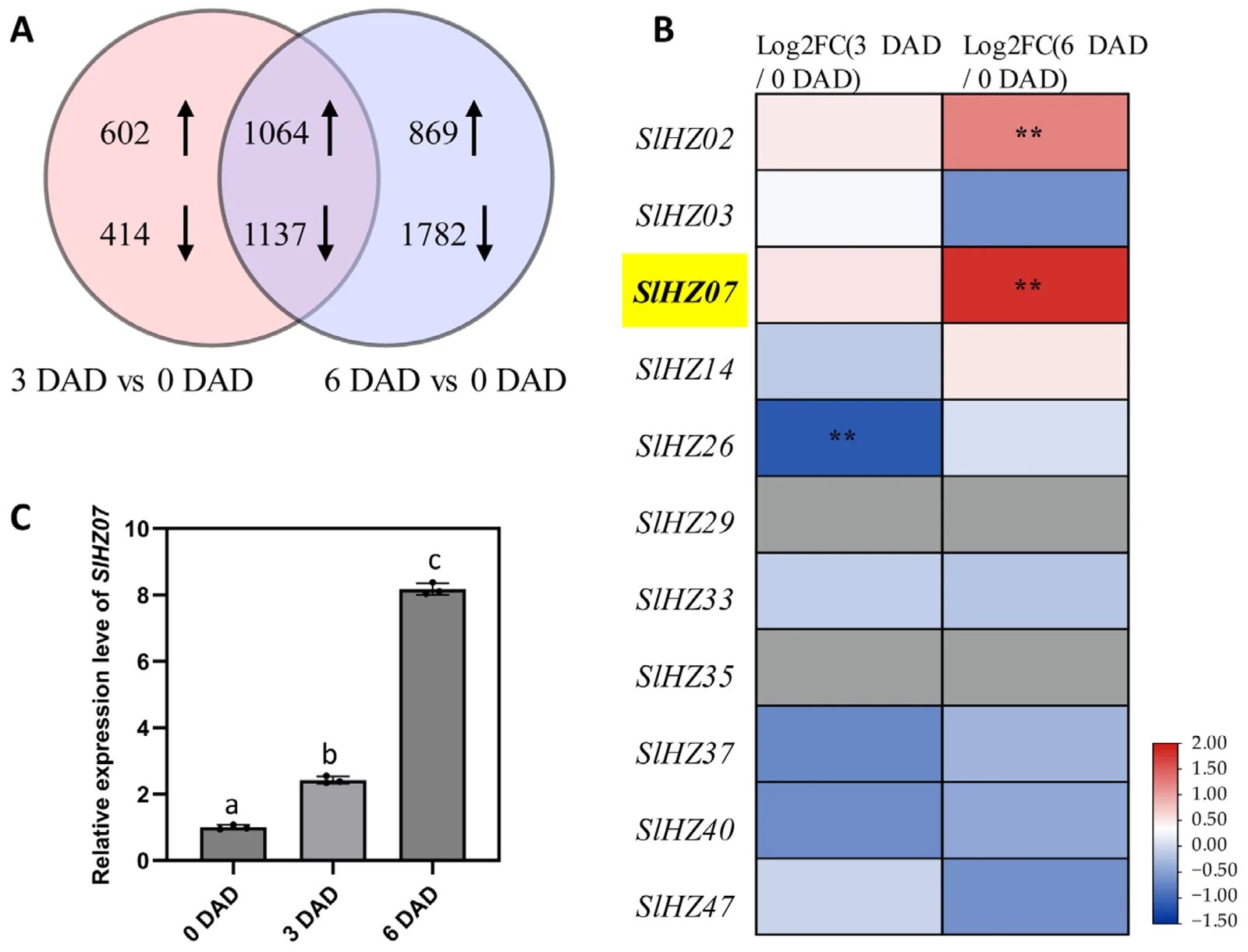
Identification of drought-responsive genes. (**A**) Venn diagram showing the numbers of DEGs in MT leaves at 3 and 6 days after drought (DAD) treatment relative to the well-watered control (0 DAD). Upward arrows indicate upregulation, and downward arrows indicate downregulation. (**B**) Heatmap showing the expression profiles of HD-Zip II TFs in MT leaves at 0, 3, and 6 DAD. Color scale represents log_2_ fold change (Log2FC) relative to 0 DAD. Asterisks indicate DEGs (fold change ≥ 2, adjusted *p*-value < 0.05). (**C**) Relative transcript levels of *SlHZ07* in MT leaves at 0, 3, and 6 DAD, determined by qRT-PCR. Data are presented as the mean ± SD (*n* = 3 biological replicates). Letters indicate significant differences between means (*p* < 0.05, one-way ANOVA).

To identify genes responding to drought stress, one-month-old MT plants were subjected to drought treatment. RNA-seq analysis of tomato leaves detected the expression of 34,593 genes. As shown in Figure 1A, compared with the control, 1666 genes were upregulated and 1551 genes were downregulated after 3 days of drought stress. After 6 days of drought stress, 1933 genes were upregulated and 2919 genes were downregulated relative to the control. By comparing the two time points, 1064 genes were identified as commonly upregulated and 1137 genes as commonly downregulated under both 3-day and 6-day drought stress compared with the control, indicating that these genes are drought-responsive.

In this study, we specifically focused on the HD-Zip II family, which has been reported to play crucial roles in the regulation of stress responses and tolerance [22,34]. The tomato genome contains 11 HD-Zip II members, of which nine were detected by transcriptomic analysis (Figure 1B). *SlHZ02*, *SlHZ07*, and *SlHZ26* were identified as DEGs (Figure 1B). Notably, among these drought-responsive genes, *SlHZ07* attracted particular attention, as it exhibited the highest level of induction among HD-Zip II members. The drought-induced expression of *SlHZ07* was further validated by qRT-PCR. *SlHZ07* expression level was significantly upregulated at 3 DAD and further increased at 6 DAD (Figure 1C). Taken together, these results demonstrate that the expression of the tomato HD-Zip II TF *SlHZ07* is positively and actively responsive to drought stress.

### 3.2. *SlHZ07* Enhances Drought Tolerance in Tomato

*SlHZ07* is an HD-Zip-II TF whose biological function has not yet been characterized. To investigate the subcellular localization of *SlHZ07*, MT plants were transformed with a *35S-SlHZ07-GFP* construct. Confocal laser scanning microscopy revealed that *SlHZ07*-GFP fluorescence was predominantly localized to the nucleus, as indicated by strong co-localization with DAPI-stained nuclei, consistent with its predicted function as a TF (Figure S1A). In addition, the spatial expression pattern of *SlHZ07* was examined across different tissues. *SlHZ07* transcripts accumulated predominantly in roots and in fruits at the red-ripe stage, whereas lower expression levels were detected in leaves (Figure S1B).

**Figure 2:**
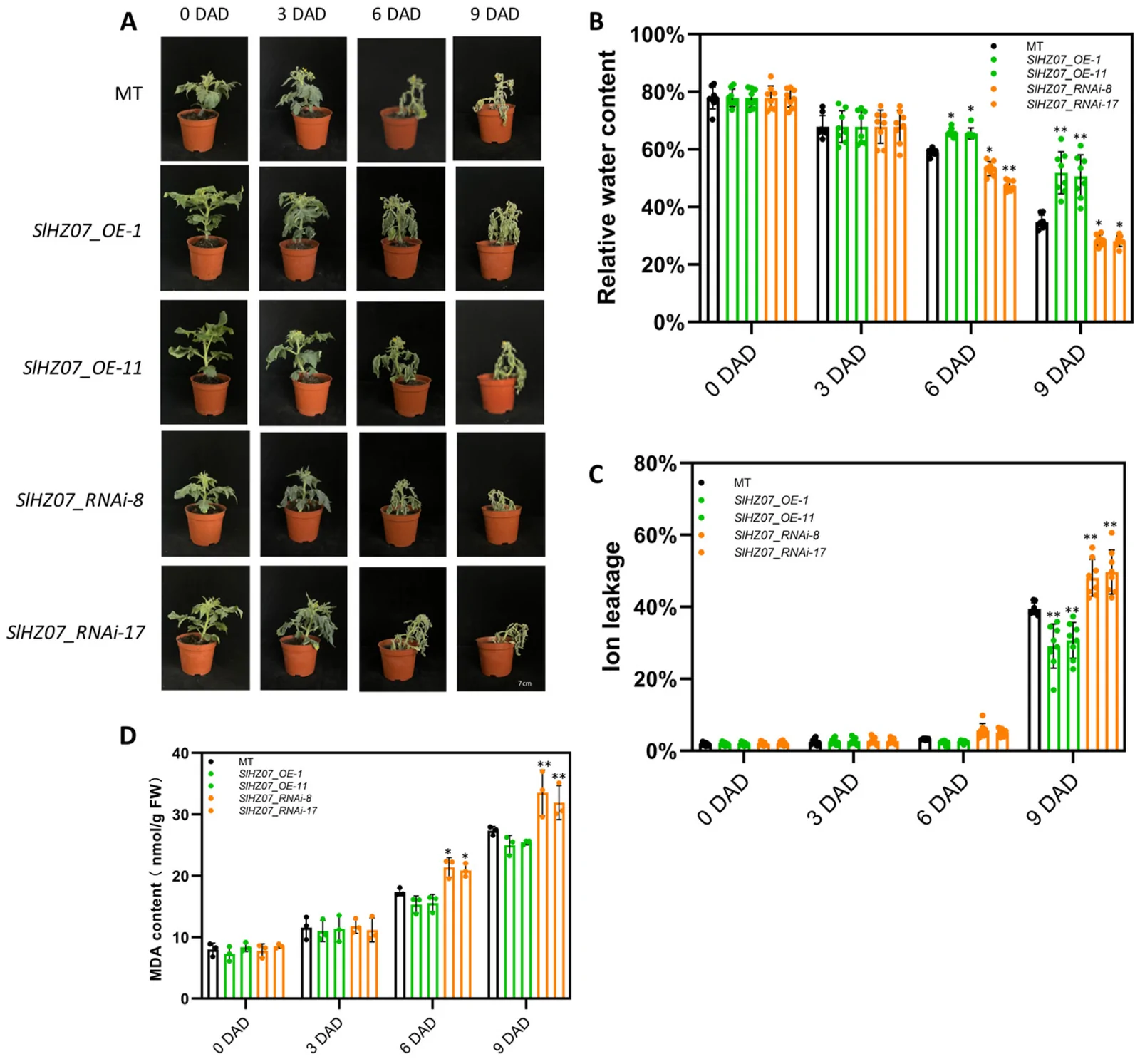
The response of MT, *SlHZ07_OE* and *SlHZ07_RNAi* plants to drought stress. (**A**) Representative phenotypes of MT, *SlHZ07_OE* and *SlHZ07_RNAi* plants at 0, 3, 6, and 9 DAD. (**B**–**D**) RWC (**B**), ion leakage (**C**), and MDA content (**D**) in leaves of MT, *SlHZ07_OE* and *SlHZ07_RNAi* plants at 0, 3, 6 and 9 DAD. Data represent mean ± SD (*n* = 3 biological replicates). Asterisks indicate significant differences relative to MT (* *p* < 0.05, ** *p* < 0.01; two-way ANOVA).

To further characterize the biological functions of *SlHZ07*, MT plants overexpressing *SlHZ07* and *SlHZ07* RNAi knockdown lines were generated. Altered *SlHZ07* transcript abundance in these transgenic lines was verified by qRT-PCR, and representative lines with significantly increased or reduced expression were selected for further analysis (Figure S2). Given that *SlHZ07* is a drought-responsive gene, we next investigated its role in drought tolerance by subjecting six-week-old plants to drought stress. Under water-deficit conditions, *SlHZ07_OE* lines (*SlHZ07_OE-1* and *SlHZ07_OE-11*) displayed enhanced drought tolerance, exhibiting improved survival and reduced wilting compared with MT plants at 6 and 9 DAD (Figure 2A). In contrast, *SlHZ07_RNAi* lines (*SlHZ07_RNAi-8* and *SlHZ07_RNAi-17*) showed pronounced wilting symptoms, particularly at 9 DAD.

Physiological measurements supported these observations. RWC was significantly higher in *SlHZ07_OE* lines than in MT plants at both 6 and 9 DAD, whereas *SlHZ07_RNAi* lines exhibited significantly lower RWC (Figure 2B). Consistently, ion leakage was significantly lower in *SlHZ07_OE* leaves compared with MT at 9 DAD, indicating decreased membrane damage under drought stress (Figure 2C). Conversely, *SlHZ07_RNAi* plants showed significantly elevated ion leakage at both 6 and 9 DAD (Figure 2C). In addition, MDA content was significantly higher in *SlHZ07_RNAi* leaves than in MT at both 6 and 9 DAD (Figure 2D), indicating more severe membrane lipid peroxidation under drought stress. Together, these results demonstrate that *SlHZ07* positively regulates drought tolerance in tomato.

### 3.3. *SlHZ07* Modulates ROS Homeostasis Under Drought Stress

**Figure 3:**
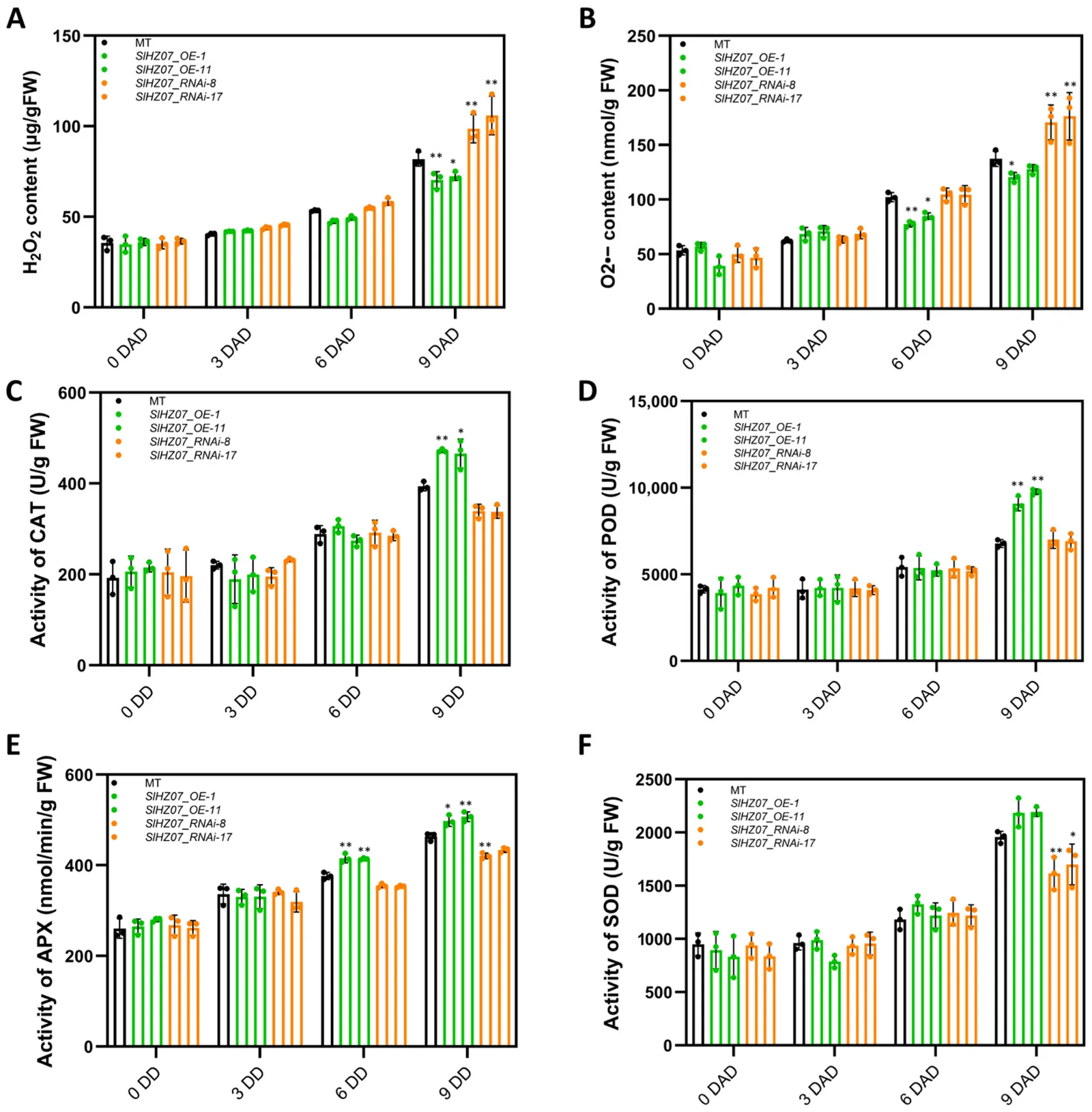
Altered *SlHZ07* expression affects ROS accumulation and antioxidant enzyme activities under drought stress. (**A**–**F**), H_2_O_2_ content (**A**), O_2_•^−^ content (**B**), and the activities of CAT (**C**), POD (**D**), APX (**E**), and SOD (**F**) in leaves of MT, *SlHZ07_OE* and *SlHZ07_RNAi* plants at 0, 3, 6 and 9 DAD. Data represent mean ± SD (*n* = 3 biological replicates). Asterisks indicate significant differences relative to MT (* *p* < 0.05, ** *p* < 0.01; two-way ANOVA).

Previous studies revealed that drought stress triggers a burst of ROS in plants [9,12]. To investigate whether *SlHZ07* enhances drought tolerance in tomato by modulating ROS accumulation, we measured the levels of H_2_O_2_ and O_2_•^−^ (Figure 3A,B). Compared with MT, *SlHZ07-RNAi* leaves accumulated significantly higher levels of H_2_O_2_ and O_2_•^−^ at 9 DAD, whereas *SlHZ07_OE* leaves showed reduced accumulation of both ROS. Notably, a decrease in O_2_•^−^ level was already observed in *SlHZ07_OE* leaves at 6 DAD, suggesting that *SlHZ07* suppresses drought-induced ROS burst in tomato leaves. We further assessed the activities of antioxidant enzymes in MT, *SlHZ07_RNAi*, and *SlHZ07_OE* plants. As shown in Figure 3C–F, the activities of CAT, POD, APX, and SOD were comparable among genotypes at 0 and 3 DAD. However, at 6 and 9 DAD, APX activity was significantly higher in *SlHZ07_OE* lines than in MT. In addition, CAT, SOD, and POD activities were also significantly elevated in *SlHZ07_OE* leaves than in MT at 9 DAD, whereas APX and SOD activities were significantly lower in *SlHZ07_ RNAi* leaves than in MT.

**Figure 4:**
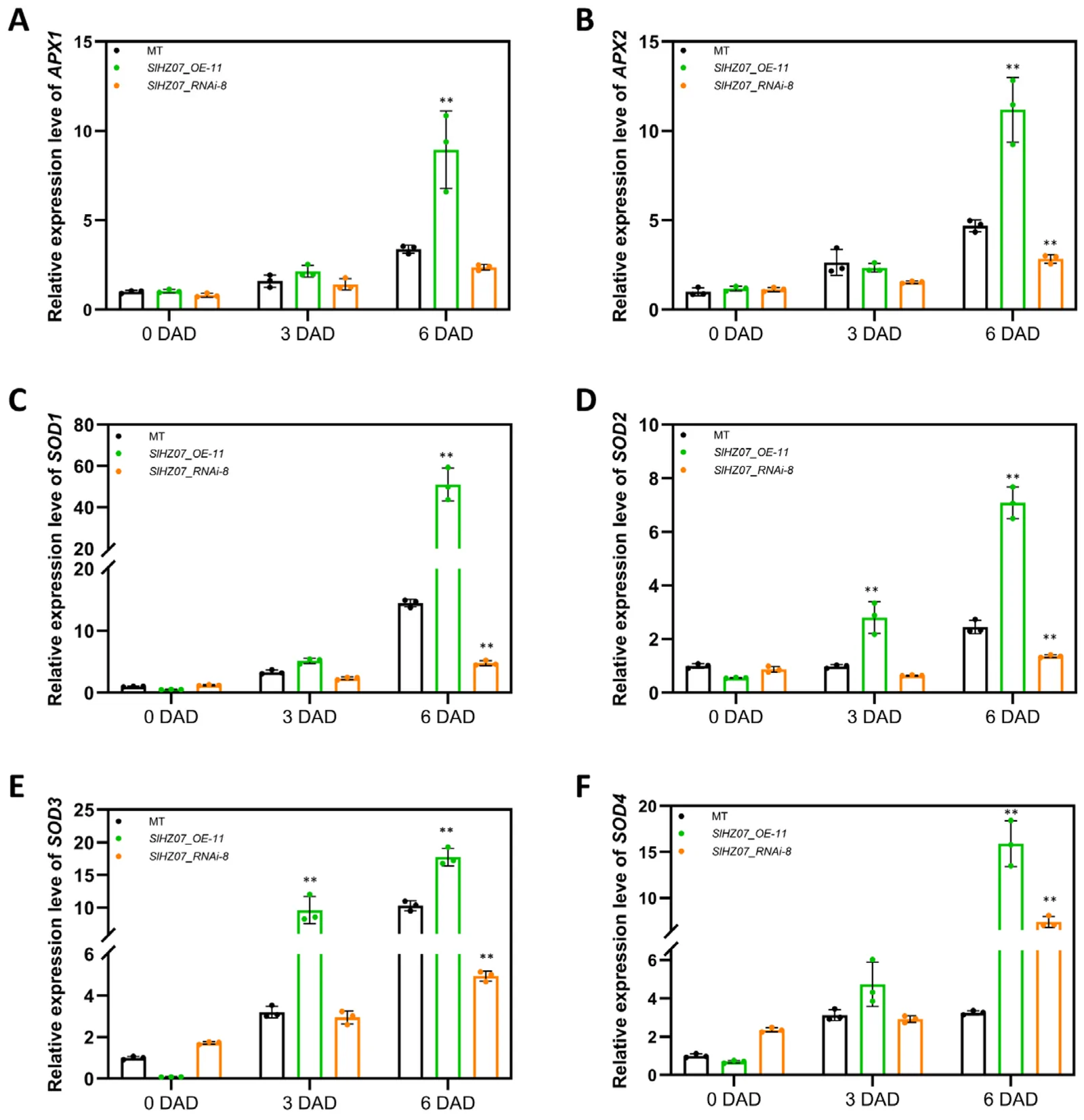
*SlHZ07* modulates antioxidant gene expression under drought stress. A-F, Relative expression levels of *APX1* (**A**), *APX2* (**B**), *SOD1* (**C**), *SOD2* (**D**), *SOD3* (**E**), and *SOD4* (**F**) in leaves of MT, *SlHZ07_OE-11* and *SlHZ07_RNAi-8* plants at 0, 3, and 6 DAD. Data represent mean ± SD (*n* = 3 biological replicates). Asterisks indicate significant differences relative to MT (** *p* < 0.01; two-way ANOVA).

Moreover, we analyzed the expression levels of several ROS-scavenging genes by qRT–PCR. The results showed that the transcript levels of *APX1*, *APX2*, *SOD1*, *SOD2*, *SOD3*, and *SOD4* were significantly higher in *SlHZ07_OE* leaves than in MT at 9 DAD (Figure 4). Notably, *SOD2* and *SOD3* were also significantly upregulated in *SlHZ07_OE* leaves at 6 DAD (Figure 4D,E). In contrast, the expression levels of *APX2*, *SOD1*, *SOD2*, *SOD3*, and *SOD4* were significantly lower in *SlHZ07_RNAi* leaves than in MT at 9 DAD (Figure 4B–F). Taken together, these results indicate that *SlHZ07* enhances drought tolerance by limiting ROS accumulation, likely through positive regulation of ROS-scavenging gene expression and maintenance of antioxidant enzyme activities under drought stress.

### 3.4. *SlHZ07* Positively Regulates JA Biosynthesis

ABA is a key phytohormone involved in plant responses to drought stress [21,35]. To determine whether *SlHZ07* enhances drought tolerance through modulation of the ABA signaling pathway, we first measured endogenous ABA levels in MT, *SlHZ07_RNAi*, and *SlHZ07_OE* plants grown under control conditions. ABA contents were comparable among these lines, suggesting that altered *SlHZ07* expression does not affect ABA accumulation (Figure S3A). We next analyzed its expression in response to ABA treatment. As shown in Figure S3B, qRT–PCR analysis revealed dynamic changes in *SlHZ07* expression in detached tomato leaves after ABA application. Specifically, *SlHZ07* expression decreased at 1 h, increased at 3 h, and returned to control levels by 5 h after treatment. These results indicate that *SlHZ07* is an ABA responsive gene which acts downstream of the ABA signaling pathway instead of a regulator of ABA biosynthesis.

**Figure 5:**
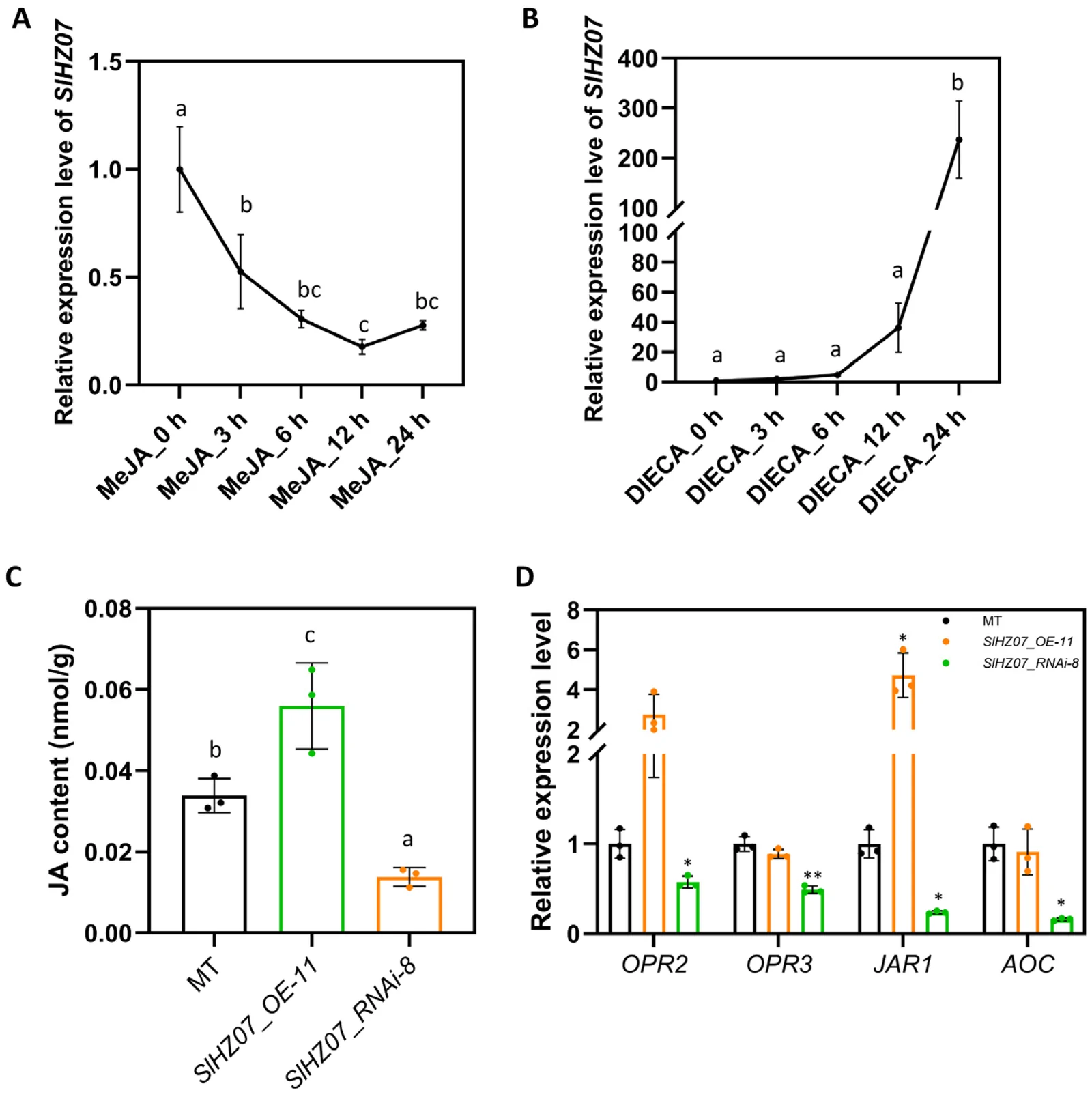
*SlHZ07* regulates JA biosynthesis. (**A**,**B**), Relative expression level of *SlHZ07* in detached 4th leaves of one-month-old MT plants following treatment with 100 μM methyl jasmonate (MeJA) (**A**) or 2 mM DIECA, a JA biosynthesis inhibitor (**B**), for 0, 3, 6, 12, and 24 h. (**C**) Endogenous JA content in leaves of MT, *SlHZ07_OE-11* and *SlHZ07_RNAi-8* at six weeks after germination. (**D**) Relative expression levels of JA biosynthetic genes in leaves of MT, *SlHZ07_OE-11* and *SlHZ07_RNAi-8* at six weeks after germination. Data are presented as the mean ± SD (*n* = 3 biological replicates). Letters indicate significant differences between means in (**A**–**C**) (*p* < 0.05; one-way ANOVA). Asterisks denote significant differences from MT (* *p* < 0.05, ** *p* < 0.01; Student’s *t*-test).

In addition to ABA, JA has also been implicated in drought responses [10]. Interestingly, *SlHZ07* expression was suppressed in MT leaves following JA treatment, whereas it was induced by the JA biosynthesis inhibitor sodium diethyldithiocarbamate (DIECA) (Figure 5A,B). Quantification of JA levels showed that *SlHZ07_OE* plants accumulated significantly higher JA levels than MT, whereas JA content was markedly reduced in *SlHZ07_RNAi* plants grown under control conditions (Figure 5C). We further examined the expression of key JA biosynthesis genes, including *OPR2*, *OPR3*, *JAR1*, and *AOC* in leaves of MT, *SlHZ07_RNAi*, and *SlHZ07_OE*. qRT–PCR analysis revealed that *OPR2*, *OPR3*, *JAR1*, and *AOC* were significantly downregulated in *SlHZ07_RNAi* plants, whereas *JAR1* expression was significantly upregulated in *SlHZ07_OE* plants (Figure 5D). Taken together, these results indicate that *SlHZ07* positively regulates JA biosynthesis, which may contribute to enhanced drought tolerance in tomato.

### 3.5. *SlHZ07* Promotes Plant Growth and GA Biosynthesis in Tomato

**Figure 6:**
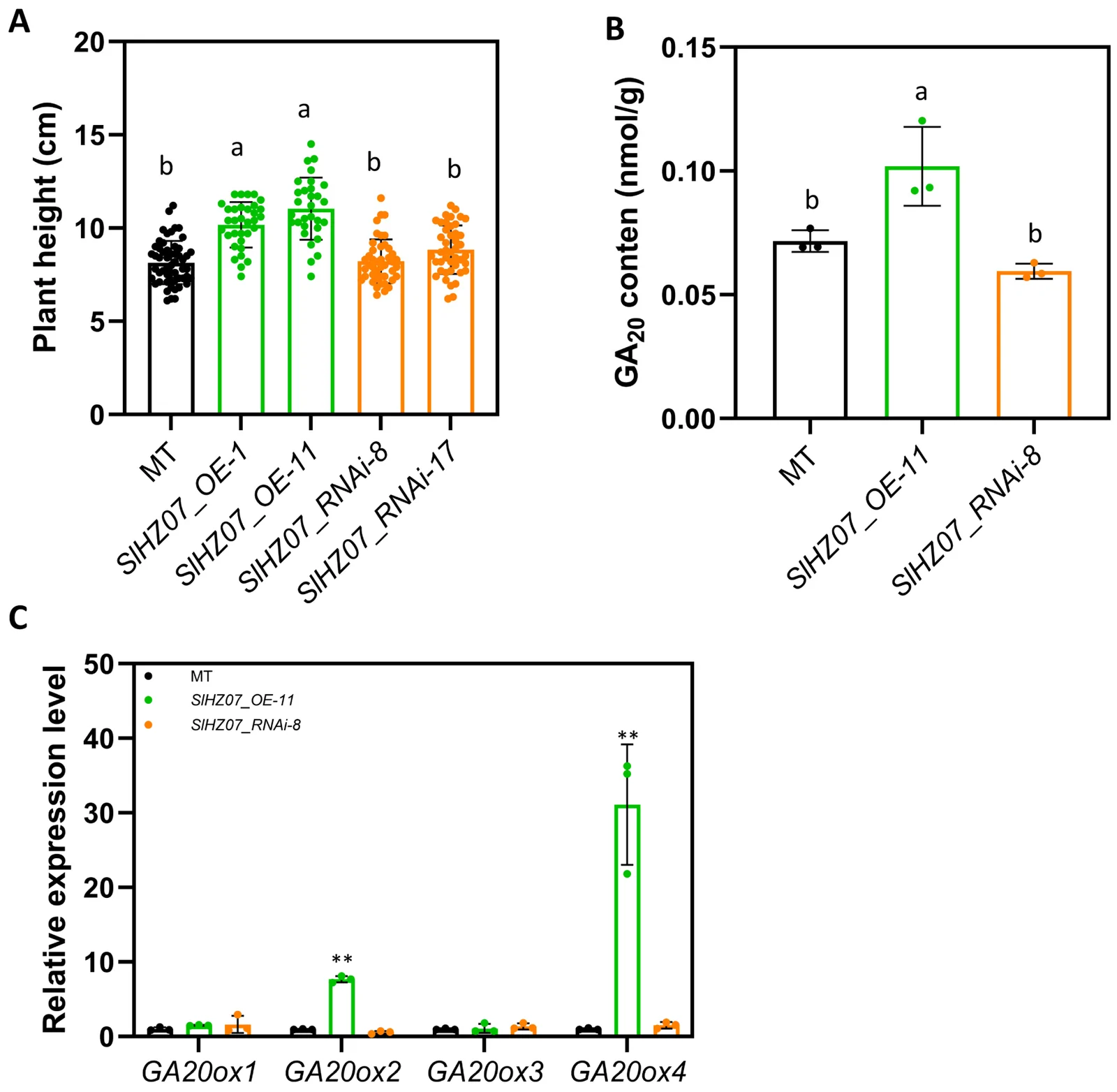
*SlHZ07* promotes stem elongation and GA biosynthesis. (**A**) Plant height of MT, *SlHZ07_OE* and *SlHZ07_RNAi* plants measured at six weeks after germination. (**B**) Endogenous GA_20_ content in leaves of MT, *SlHZ07_OE-11* and *SlHZ07_RNAi-8* at six weeks after germination. (**C**) Relative expression levels of GA20 biosynthesis genes in leaves of MT, *SlHZ07_OE-11* and *SlHZ07_RNAi-8* at six weeks after germination. Data are presented as the mean ± SD (*n* = 30–55 in (**A**); *n* = 3 in (**B**,**C**). In (**A**,**B**), letters indicate significant differences among genotypes (*p* < 0.05; one-way ANOVA). In (**C**), asterisks denote significant differences from MT (** *p* < 0.01, Student’s *t*-test).

In addition to evaluating drought responses, we compared the growth of transgenic and MT plants under well-watered conditions. As *SlHZ07* was highly expressed in tomato roots (Figure S1B), we first compared root growth among MT, *SlHZ07_OE*, and *SlHZ07_RNAi* seedlings. As shown in Figure S4, no significant differences in primary root length were observed between MT and either of the *SlHZ07* transgenic lines under control conditions. We next assessed shoot growth at six weeks after germination. Both *SlHZ07_OE* lines were significantly taller than MT plants, whereas plant height did not differ significantly between *SlHZ07_RNAi* and MT plants (Figure 6A), indicating that overexpressing *SlHZ07* promotes elongation growth in tomato. Because gibberellins are key regulators of stem growth, we next investigated whether this phenotype was associated with altered GA biosynthesis. As shown in Figure 6B, the endogenous level of the GA intermediate GA_20_ was significantly higher in *SlHZ07_OE* plants than in MT, whereas no significant difference was detected between MT and *SlHZ07_RNAi* plants. Consistent with these observations, qRT-PCR analysis showed that the GA_20_ biosynthetic genes *GA20ox2* and *GA20ox4* were significantly upregulated in *SlHZ07_OE* plants relative to MT, while the expression of *GA20ox1* and *GA20ox3* was not markedly affected (Figure 6C). Together, these results indicate that *SlHZ07* promotes stem elongation, at least in part, by enhancing GA biosynthesis in tomato.

## 4. Discussion

Drought is one of the most severe abiotic stresses limiting plant growth, crop productivity, and agricultural sustainability worldwide [8]. The increasing frequency and intensity of drought events associated with global climate change have further intensified the need to develop crop varieties with improved drought tolerance [3,11]. However, enhancing drought tolerance without compromising plant growth remains a major challenge in crop improvement. Therefore, elucidating the molecular mechanisms that coordinate plant growth and drought adaptation is of both fundamental biological and agronomic importance. HD-Zip TFs have emerged as important regulators of plant development and stress responses [34], yet the functions of most HD-Zip II members in tomato remain largely unexplored. Therefore, this study was performed to characterize the biological function of the drought-responsive HD-Zip II gene *SlHZ07* and to elucidate its role in regulating tomato growth and drought tolerance.

### 4.1. *SlHZ07* Is a Drought-Responsive HD-Zip II TF That Coordinates Plant Growth and Drought Adaptation

Plants continuously balance growth and stress adaptation to maximize fitness under fluctuating environmental conditions. Activation of stress-responsive pathways often enhances tolerance at the expense of growth because metabolic resources are preferentially allocated to defense rather than biomass accumulation [20,36]. Consequently, TFs that simultaneously promote plant growth and drought tolerance are of particular interest for crop improvement.

The plant-specific HD-Zip TFs play diverse roles in development and environmental adaptation [15,17,37]. Although HD-Zip I proteins have been extensively implicated in drought responses in several plant species [34], comparatively little is known about the physiological functions of HD-Zip II members, particularly in tomato. Our previous genome-wide analyses identified *SlHZ07* as one of the most dehydration-responsive HD-Zip II genes [23], but its biological function remained unknown. Here, transcriptomic and qRT-PCR analyses consistently demonstrated that *SlHZ07* is rapidly induced during drought stress (Figure 1B,C), supporting its involvement in the early transcriptional response to water deficit. The predominant nuclear localization of *SlHZ07* protein further supports its role as a transcriptional regulator (Figure S1A). Together, these observations suggest that *SlHZ07* functions as a component of the drought-responsive transcriptional network in tomato.

A notable finding of this study is that overexpression of *SlHZ07* markedly enhanced drought tolerance while simultaneously promoting vegetative growth (Figure 2 and Figure 6). Although *SlHZ07* was highly expressed in roots (Figure S1B), we detected no significant differences in primary root length between MT and *SlHZ07* transgenic plants under control conditions (Figure S4), suggesting that the enhanced growth phenotype of the transgenic plants is unlikely to result from altered primary root elongation under control conditions. Whether *SlHZ07* affects root growth under drought conditions and whether other components of root architecture contribute to the enhanced drought tolerance of *SlHZ07_OE* plants remain to be investigated. The simultaneous enhancement of growth and drought tolerance contrasts with many previously characterized drought-responsive TFs, whose constitutive activation frequently causes dwarfism or growth inhibition owing to excessive activation of stress signaling pathways [11,20,38]. The ability of *SlHZ07* to improve drought tolerance without an obvious growth penalty suggests that it coordinates growth and stress adaptation rather than prioritizing one process over the other.

Consistent with this hypothesis, *SlHZ07_OE* plants exhibited increased endogenous GA_20_ accumulation together with elevated expression of *GA20ox2* and *GA20ox4* (Figure 6C), indicating enhanced GA biosynthetic activity. GA is a major regulator of stem elongation and biomass accumulation, yet enhanced GA signaling is often considered incompatible with drought adaptation because GA generally promotes growth at the expense of stress tolerance [39,40,41]. Whether *SlHZ07* directly regulates GA biosynthetic genes or functions through additional transcriptional regulators remains to be determined.

### 4.2. *SlHZ07* Enhances Drought Tolerance by Maintaining ROS Homeostasis

Oxidative damage is a major consequence of drought stress [6]. Water deficit disrupts cellular redox homeostasis, leading to excessive accumulation of ROS, including H_2_O_2_ and O_2_•^−^, which damage membrane lipids, proteins, and nucleic acids [8,9]. Maintenance of ROS homeostasis therefore represents a fundamental mechanism underlying drought tolerance [6,13]. Our physiological analyses demonstrated that *SlHZ07_OE* plants accumulated lower levels of H_2_O_2_ and O_2_•^−^, exhibited reduced membrane lipid peroxidation and ion leakage, and maintained higher RWC during drought stress (Figure 2 and Figure 3). These physiological improvements were accompanied by increased activities of CAT, POD, APX, and SOD, together with elevated expression of multiple ROS-scavenging genes (Figure 4). These findings indicate that *SlHZ07* enhances drought tolerance primarily by strengthening the antioxidant defense system and limiting oxidative damage under prolonged water deficit. Similar mechanisms have been reported for other stress-responsive TFs [36,42,43,44]; however, our results establish HD-Zip II proteins as additional regulators of ROS homeostasis in tomato.

### 4.3. *SlHZ07* Integrates JA Signaling with Drought Tolerance but Functions Independently of ABA Biosynthesis

Phytohormones play central roles in coordinating plant responses to environmental stress [10,21]. ABA has long been regarded as the master regulator of drought responses, whereas the contribution of JA has received increasing attention in recent years [10,45,46]. Interestingly, although *SlHZ07* expression responded dynamically to exogenous ABA treatment (Figure S3B), endogenous ABA levels remained unchanged in both overexpression and RNAi lines under control conditions (Figure S3A). These observations suggest that *SlHZ07* functions downstream of ABA perception rather than directly regulating ABA biosynthesis under control conditions. In contrast, *SlHZ07* clearly influenced JA accumulation under control conditions. Overexpression of *SlHZ07* increased endogenous JA accumulation and enhanced the expression of several JA biosynthetic genes, whereas *SlHZ07_RNAi* lines displayed the opposite trends (Figure 5C,D). These results support a positive role for *SlHZ07* in promoting JA biosynthesis. Whether *SlHZ07* similarly affects JA accumulation under drought conditions remains to be determined and represents an important direction for future research.

HD-Zip II TFs are known to recognize pseudopalindromic DNA motifs containing the consensus sequence CAAT(C/G)ATTG [17,37]. Because the JA biosynthetic gene *JAR1* was significantly upregulated in *SlHZ07_OE* plants and down regulated in *SlHZ07_RNAi* plants (Figure 5D), we examined whether *JAR1* is a direct transcriptional target of *SlHZ07*. Sequence analysis identified a putative HD-Zip II binding site located 184 bp upstream of the *JAR1* transcription start site. A 550 bp fragment of the *JAR1* promoter containing this motif was examined by Y1H assay (Figure S5). However, *SlHZ07* did not bind the tested *JAR1* promoter fragment, suggesting that the altered *JAR1* expression observed in the transgenic lines is unlikely to result from direct binding of *SlHZ07* to the tested promoter region. Instead, *SlHZ07* may regulate other components of JA metabolism or signaling that subsequently affect *JAR1* expression. It is also possible that regulatory elements located outside the tested promoter region contribute to *JAR1* regulation. Further studies are required to identify the molecular components linking *SlHZ07* to JA homeostasis. In addition, HD-Zip TFs frequently function within complex transcriptional regulatory networks to control downstream gene expression [16,27,34]. Elucidating the direct downstream target genes of *SlHZ07* and the molecular mechanisms by which it coordinates hormone signaling and antioxidant responses will therefore be important objectives for future studies.

**Figure 7:**
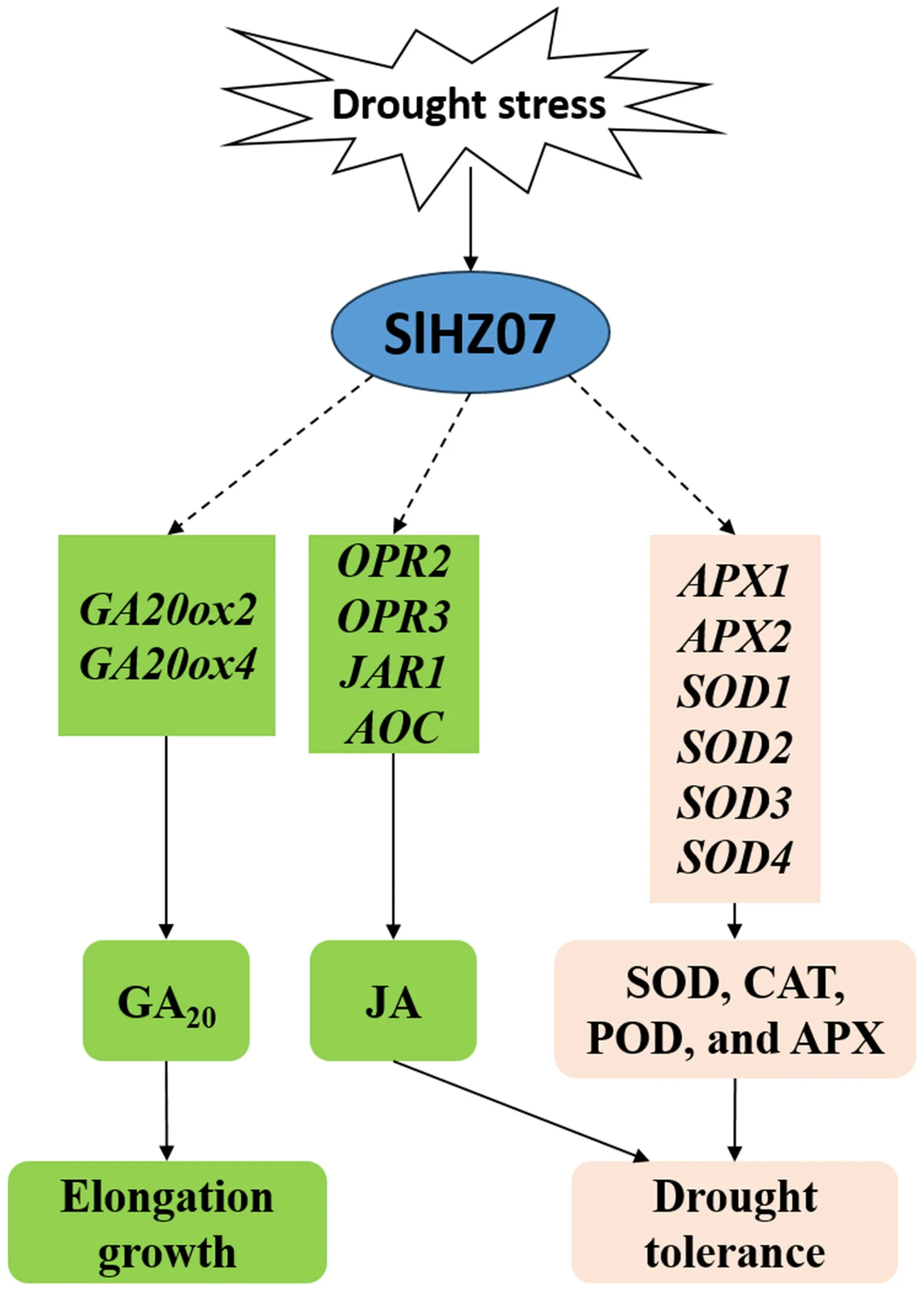
Proposed model illustrating the role of the HD-Zip II TF *SlHZ07* in coordinating plant growth and drought adaptation in tomato. Under control conditions, *SlHZ07* promotes GA_20_ biosynthesis by upregulating the expression of *GA20ox2* and *GA20ox4*, resulting in increased endogenous GA_20_ levels and enhanced vegetative growth. In parallel, *SlHZ07* positively regulates JA accumulation and the expression of JA biosynthetic genes (*OPR2*, *OPR3*, *JAR1*, and *AOC*), thereby contributing to drought adaptation. Under drought conditions, *SlHZ07* expression is induced and enhances antioxidant capacity by promoting the expression of ROS-scavenging genes and increasing the activities of antioxidant enzymes, including SOD, CAT, POD, and APX. Consequently, ROS accumulation, membrane lipid peroxidation, and cellular membrane damage are reduced under drought stress, thereby enhancing drought tolerance. Solid arrows indicate direct regulatory relationships supported by experimental evidence, whereas dashed arrows indicate indirect regulatory relationships or relationships for which direct regulation has not yet been experimentally demonstrated.

Collectively, our findings suggest that *SlHZ07* partially alleviates the classical trade-off between growth and stress tolerance by promoting GA and JA biosynthesis while enhancing antioxidant defenses (Figure 7). Although the controlled drought treatment used in this study effectively revealed the role of *SlHZ07* in drought tolerance, plant responses under field conditions are likely to be influenced by additional environmental factors, including interactions with soil microorganisms [47,48,49]. Rhizosphere microorganisms can produce, degrade, or otherwise modulate phytohormones, including GA and JA, and may thereby influence plant growth and stress responses [48,50,51]. Future field experiments integrating rhizosphere microbial community profiling with analyses of hormone metabolism will therefore be important for determining whether plant–microbe interactions modify the *SlHZ07*-mediated drought response and for further evaluating its physiological and agronomic significance.

## 5. Conclusions

In summary, this study demonstrates that the HD-Zip II TF *SlHZ07* functions as a positive regulator of both plant growth and drought tolerance in tomato. *SlHZ07* is rapidly induced by drought stress and enhances drought tolerance by promoting antioxidant defenses, maintaining ROS homeostasis, and positively regulating JA accumulation, while simultaneously stimulating GA biosynthesis to support vegetative growth. These findings suggest that *SlHZ07* partially alleviates the classical trade-off between growth and stress adaptation by coordinating multiple physiological and hormonal pathways. Although the direct downstream targets of *SlHZ07* remain to be identified, our work establishes *SlHZ07* as a key component of the drought-responsive regulatory network in tomato and provides new insights into the biological functions of HD-Zip II TFs. This study also identifies *SlHZ07* as a promising genetic resource for improving drought tolerance while maintaining plant growth in tomato breeding programs.

## Data Availability

Data is contained within the article.

## Supplementary Materials

The following supporting information can be downloaded at https://www.mdpi.com/article/10.3390/antiox15091062/s1, Figure S1: Subcellular localization and tissue-specific expression of *SlHZ07*. (A) Confocal microscope analysis showing nuclear localization of *SlHZ07*-GFP and DAPI stained nucleus in leaf cells. BF, bright field. Scale bar, 25 μm. (B) Relative expression level of *SlHZ07* in different tissues of MT, including roots, stems, leaves, flowers, and fruits at the mature green (MG), breaker (Br), orange (Or), and red-ripe (RR) stages. Data represent mean ± SD (*n* = 3 biological replicates); Figure S2: Expression levels of *SlHZ07* in leaves of one month old seedling of *SlHZ07_OE* and *SlHZ07_RNAi* lines compared to MT. Data are presented as the mean ± SD (*n* = 3 biological replicates). Asterisks denote significant differences from MT (** *p* < 0.01, Student’s *t*-test); Figure S3: Relationship between *SlHZ07* expression and ABA. (A) Endogenous ABA content in leaves of MT, *SlHZ07_OE-11* and *SlHZ07_RNAi-8* at six weeks after germination. (B) Relative expression level of *SlHZ07* in detached 4th leaves of one-month-old MT plants following treatment with 100 μM ABA for 0, 1, 3, and 5 h. Data are presented as the mean ± SD (*n* = 3 biological replicates). Different letters indicate significant differences among treatments or genotypes (*p* < 0.05; one-way ANOVA); Figure S4: Root growth of MT, *SlHZ07_OE-11*, and *SlHZ07_RNAi-8* seedlings under control conditions. (A) Representative images of MT, *SlHZ07_OE-11*, and *SlHZ07_RNAi-8* seedlings grown on MS medium for 20 days after germination. (B) Primary root length of the indicated genotypes. Data are presented as means ± SD (*n* = 10 biological replicates); Figure S5: *SlHZ07* does not bind to the *JAR1* promoter in the Y1H assay. A 550 bp *JAR1* promoter fragment containing the predicted HD-Zip II binding site was cloned into the pAbAi vector to generate the bait construct (*pAbAi-JAR1pro*). Yeast cells co-transformed with *pAbAi-JAR1pro* and *pGADT7-SlHZ07-AD* vectors failed to grow on SD/-Leu selective medium supplemented with 50 ng/mL aureobasidin A (AbA). Table S1: Primers for cloning; Table S2: qRT-PCR primers.

## References

[1] Li Y., Henke M., Zhang D., Wang C., Wei M. (2024). Optimized tomato production in Chinese solar greenhouses: The impact of an east–west orientation and wide row spacing. Agronomy.

[2] Wang S., Qiang Q., Xiang L., Fernie A.R., Yang J. (2023). Targeted approaches to improve tomato fruit taste. Hortic. Res..

[3] Ma L., Wang H., Zhang B., Guo Y., Ou S., Tian Y., Guo M. (2026). Integrated strategies for enhanced drought tolerance in tomato: From molecular mechanisms to field applications. Hortic. Res..

[4] Chowdhury R.H., Eti F.S., Ahmed R., Gupta S.D., Jhan P.K., Islam T., Bhuiyan M.A.R., Rubel M.H., Khayer A. (2023). Drought-responsive genes in tomato: Meta-analysis of gene expression using machine learning. Sci. Rep..

[5] Mukherjee S., Dash P.K., Das D., Das S. (2023). Growth, yield and water productivity of tomato as influenced by deficit irrigation water management. Environ. Process..

[6] Song F., Yang Q., Huang J., Guo Z., Li Y., Deng W. (2026). Plant drought stress: Physiological, biochemical and molecular mechanisms. Plant Stress.

[7] Qiu L., Chen X., Hou H., Fan Y., Wang L., Zeng H., Chen X., Ding Y., Hu X., Yan Q. (2023). Genome-wide characterization of the tomato UDP-glycosyltransferase gene family and functional identification of *SlUDPGT52* in drought tolerance. Hortic. Adv..

[8] Gupta A., Rico-Medina A., Caño-Delgado A.I. (2020). The physiology of plant responses to drought. Science.

[9] Patanè C., Cosentino S.L., Romano D., Toscano S. (2022). Relative water content, proline, and antioxidant enzymes in leaves of long shelf-life tomatoes under drought stress and rewatering. Plants.

[10] Zhu C., Li X., Zhang M., Wang S., Jing B., Hu C., Thomas H.R., Zhou Y., Yu J., Hu Z. (2025). ERF.D2 negatively controls drought tolerance through synergistic regulation of abscisic acid and jasmonic acid in tomato. Plant Biotechnol. J..

[11] Wei H., Wang X., Wang K., Tang X., Zhang N., Si H. (2024). Transcription factors as molecular switches regulating plant responses to drought stress. Physiol. Plant..

[12] Wang X., Wei H., Wang K., Tang X., Li S., Zhang N., Si H. (2025). MYB transcription factors: Acting as molecular switches to regulate different signaling pathways to modulate plant responses to drought stress. Ind. Crops Prod..

[13] Yang Y., Xu Y., Feng B., Li P., Li C., Zhu C.-Y., Ren S.-N., Wang H.-L. (2025). Regulatory networks of bZIPs in drought, salt and cold stress response and signaling. Plant Sci..

[14] Guo J., Sun B., He H., Zhang Y., Tian H., Wang B. (2021). Current understanding of bHLH transcription factors in plant abiotic stress tolerance. Int. J. Mol. Sci..

[15] Zhang J., Chen C., Yang Q., Xu J., Han Z., Ma W., Zhang X., Xu K., Zhao J., Chen X. (2025). Evolution of HD-ZIP transcription factors and their function in cabbage leafy head formation. Front. Plant Sci..

[16] Zheng J., Wang F., Mai Y., Su X., Xia R. (2026). HD-ZIP transcription factors: Pivotal regulators of plant reproductive development. Hortic. Plant J..

[17] Ahangarani Farahani Y.H., Norouzi M., Mohammadi S.A. (2025). Genome-wide identification and expression analysis of HD-ZIP gene family in sunflower (*Helianthus annuus* L.) under water deficit stress. Sci. Rep..

[18] Wu M., Chang J., Han X., Shen J., Yang L., Hu S., Huang B.-B., Xu H., Xu M., Wu S. (2023). A HD-ZIP transcription factor specifies fates of multicellular trichomes via dosage-dependent mechanisms in tomato. Dev. Cell.

[19] Li F., Fu M., Zhou S., Xie Q., Chen G., Chen X., Hu Z. (2023). A tomato HD-zip I transcription factor, VAHOX1, acts as a negative regulator of fruit ripening. Hortic. Res..

[20] Ebrahimian-Motlagh S., Ribone P.A., Thirumalaikumar V.P., Allu A.D., Chan R.L., Mueller-Roeber B., Balazadeh S. (2017). JUNGBRUNNEN1 confers drought tolerance downstream of the HD-Zip I transcription factor AtHB13. Front. Plant Sci..

[21] Jiao P., Jiang Z., Miao M., Wei X., Wang C., Liu S., Guan S., Ma Y. (2024). *Zmhdz9*, an HD-Zip transcription factor, promotes drought stress resistance in maize by modulating ABA and lignin accumulation. Int. J. Biol. Macromol..

[22] Zhang Z., Chen X., Guan X., Liu Y., Chen H., Wang T., Mouekouba L.D.O., Li J., Wang A. (2014). A genome-wide survey of homeodomain-leucine zipper genes and analysis of cold-responsive HD-Zip I members’ expression in tomato. Biosci. Biotechnol. Biochem..

[23] Dong S., Ling J., Song L., Zhao L., Wang Y., Zhao T. (2023). Transcriptomic profiling of tomato leaves identifies novel transcription factors responding to dehydration stress. Int. J. Mol. Sci..

[24] Hong Y., Liu Y., Zhang Y., Jia L., Yang X., Zhang X., Liu J., Luan Y. (2021). Genome-wide characterization of homeobox-leucine zipper gene family in tomato (*Solanum lycopersicum*) and functional analysis of SlHDZ34 (III sub-family member) under salinity stress. Environ. Exp. Bot..

[25] Hu T., Ye J., Tao P., Li H., Zhang J., Zhang Y., Ye Z. (2016). The tomato HD-Zip I transcription factor SlHZ24 modulates ascorbate accumulation through positive regulation of the D-mannose/L-galactose pathway. Plant J..

[26] Li X.H., Kim J.K., Park S.U. (2022). Heterologous expression of three transcription factors differently regulated astragalosides metabolic biosynthesis in *Astragalus membranaceus* hairy roots. Plants.

[27] Dong S., Tarkowska D., Sedaghatmehr M., Welsch M., Gupta S., Mueller-Roeber B., Balazadeh S. (2022). The HB40-JUB1 transcriptional regulatory network controls gibberellin homeostasis in *Arabidopsis*. Mol. Plant.

[28] Arvidsson S., Kwasniewski M., Riaño-Pachón D.M., Mueller-Roeber B. (2008). QuantPrime—A flexible tool for reliable high-throughput primer design for quantitative PCR. BMC Bioinform..

[29] Sampathkumar A., Wightman R. (2015). Live cell imaging of the cytoskeleton and cell wall enzymes in plant cells. Methods Mol. Biol..

[30] Beier S., Bolger A.M., Bolger M.E., Schwacke R., Usadel B. (2026). Trimmomatic: A decade of feature-rich, high-performance NGS read preprocessing. Bioinformatics.

[31] Zhang Y., Park C., Bennett C., Thornton M., Kim D. (2021). Rapid and accurate alignment of nucleotide conversion sequencing reads with HISAT-3N. Genome Res..

[32] Thakur V. (2024). RNA-Seq data analysis for differential gene expression using HISAT2-StringTie-Ballgown pipeline. Methods Mol. Biol..

[33] Deng L., Chen Y., An Y., Zhang F., Zhou P., Mou C., Chen Y. (2025). Integrating transcriptome and metabolome to reveal the characteristics of gene expression profiles and metabolite changes of sorghum in response to cadmium treatment. Front. Plant Sci..

[34] Li Y., Yang Z., Zhang Y., Guo J., Liu L., Wang C., Wang B., Han G. (2022). The roles of HD-ZIP proteins in plant abiotic stress tolerance. Front. Plant Sci..

[35] Wang G., Liu X., Gan S.S. (2023). The ABA-AtNAP-SAG113 PP2C module regulates leaf senescence by dephoshorylating SAG114 SnRK3.25 in *Arabidopsis*. Mol. Hortic..

[36] Hua Q., Feng Y., Zheng L., Li X., Li K., Xu H. (2025). Overexpression of *SlGRF4* positively regulates drought stress tolerance in tomato by alleviating ROS damage and increasing nitrogen signaling pathway. Plant Sci..

[37] Chen W., Yan W., Jiang K., Huang H. (2025). Molecular insights into DNA recognition by HD-Zip transcription factors. Plant Physiol..

[38] Shohat H., Eliaz N.I., Weiss D. (2021). Gibberellin in tomato: Metabolism, signaling and role in drought responses. Mol. Hortic..

[39] Wu H., Bai B., Lu X., Li H. (2023). A gibberellin-deficient maize mutant exhibits altered plant height, stem strength and drought tolerance. Plant Cell Rep..

[40] Huang J., Xie B., Xian F., Liu K., Yan Q., He Y., Zhu L., Liu W., Jiang Y., Chen Y. (2025). Gibberellin signalling mediates nucleocytoplasmic trafficking of Sucrose Synthase 1 to regulate the drought tolerance in rice. Plant Biotechnol. J..

[41] Shohat H., Illouz-Eliaz N., Kanno Y., Seo M., Weiss D. (2020). The tomato DELLA protein PROCERA promotes abscisic acid responses in guard cells by upregulating an abscisic acid transporter. Plant Physiol..

[42] Shi P.T., Zhang R., Wu X.J., Zhang Z., Li B.B., Wang S.L., Chen H., Fan X.N., Liu W.T., Wen J. (2026). A rice calmodulin-like protein OsCML27 participates in drought tolerance by fine-tuning OsRbohB-mediated ROS signaling. Sci. Adv..

[43] Samanta S., Seth C.S., Roychoudhury A. (2024). The molecular paradigm of reactive oxygen species (ROS) and reactive nitrogen species (RNS) with different phytohormone signaling pathways during drought stress in plants. Plant Physiol. Biochem..

[44] Wang Y., Hu X., Yang J., Mo Y., Hou H., Ding Y., Gong W., Zeng P., Shang C., Zhang X. (2026). Tomato SlERF26 enhances drought tolerance by regulating SlPIP2;4-mediated ROS homeostasis. Hortic. Res..

[45] Wang X., Li Q., Xie J., Huang M., Cai J., Zhou Q., Dai T., Jiang D. (2021). Abscisic acid and jasmonic acid are involved in drought priming-induced tolerance to drought in wheat. Crop J..

[46] Ahmad J., Hayat F., Khan U., Altaf M.A., Ahmad M., Akram M.T., Kandhan K., Sheteiwy M.S., Li J., Alyafei M. (2025). Jasmonic acid as a regulator in plant stress tolerance: Mechanisms and agricultural implications. J. Crop Health.

[47] Spanier N.M., Phillips R.P. (2026). Soil microbial drought history affects physiological response of select tree species to drought stress. Oecologia.

[48] Vidal C., González F., Santander C., Pérez R., Gallardo V., Santos C., Aponte H., Ruiz A., Cornejo P. (2022). Management of rhizosphere microbiota and plant production under drought stress: A comprehensive review. Plants.

[49] Taglialegna A. (2025). Microbial allies in plant defence against drought. Nat. Rev. Microbiol..

[50] Lu H., Lu C., Huang S., Liu W., Wang L., Yang C., Wang E., Li L. (2025). Rhizosphere microbes mitigate the shade avoidance responses in Arabidopsis. Cell Host Microbe.

[51] Chen Q., Song Y., An Y., Lu Y., Zhong G. (2024). Mechanisms and impact of rhizosphere microbial metabolites on crop health, traits, functional components: A comprehensive review. Molecules.

