# Supplementary material for: The HD-Zip II Transcription Factor SlHZ07 Promotes Growth and Drought Tolerance in Tomato
Source: Antioxidants (Basel). 2026 Aug 25;15(9):1062. doi: 10.3390/antiox15091062 (PMC13603686; doi:10.3390/antiox15091062)
Supplement: Supplementary file 1 [file antioxidants-15-01062-s001.zip › Supplementary Materials Figures.pdf]

Supplementary Materials

The HD-Zip II Transcription Factor SlHZ07 Promotes Growth and Drought Tolerance in Tomato

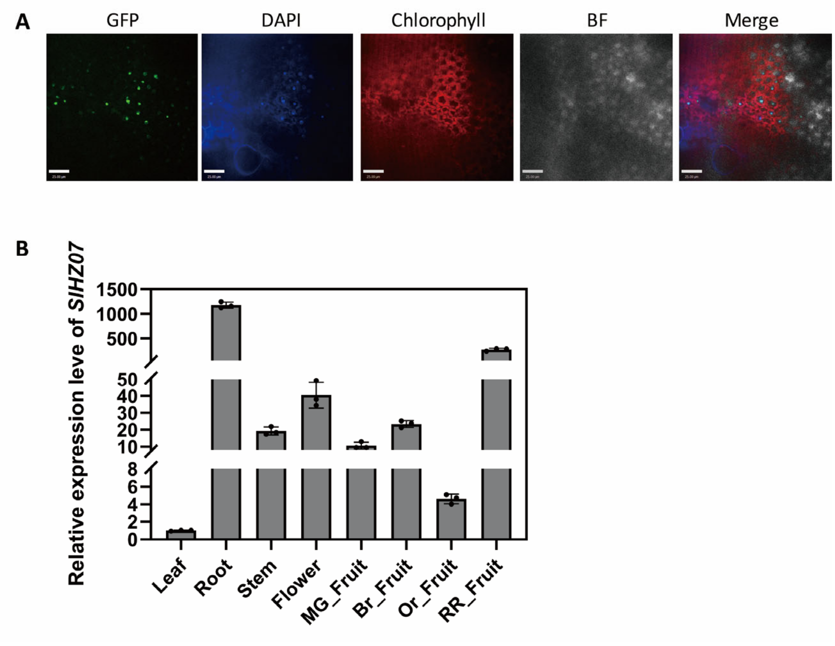

Figure S1: Subcellular localization and tissue-specific expression of *SlHZ07*. (A) Confocal microscope analysis showing nuclear localization of SlHZ07-GFP and DAPI stained nucleus in leaf cells. BF, bright field. Scale bar, 25 μm. (B) Relative expression level of *SlHZ07* in different tissues of MT, including roots, stems, leaves, flowers, and fruits at the mature green (MG), breaker (Br), orange (Or), and red-ripe (RR) stages. Data represent mean ± SD (*n* = 3 biological replicates)

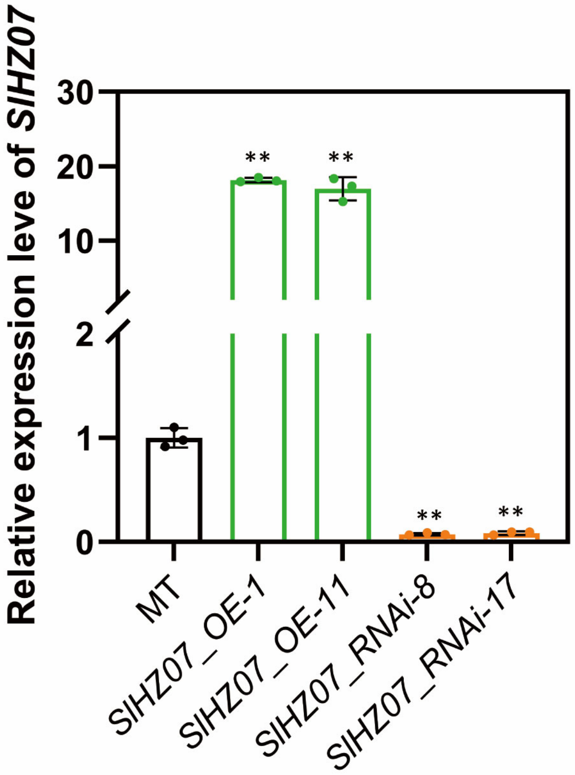

Figure S2: Expression levels of *SlHZ07* in leaves of one month old seedling of *SlHZ07_OE* and *SlHZ07_RNAi* lines compared to MT. Data are presented as the mean ± SD (n = 3 biological replicates). Asterisks denote significant differences from MT (** $p < 0.01$, Student's t-test)

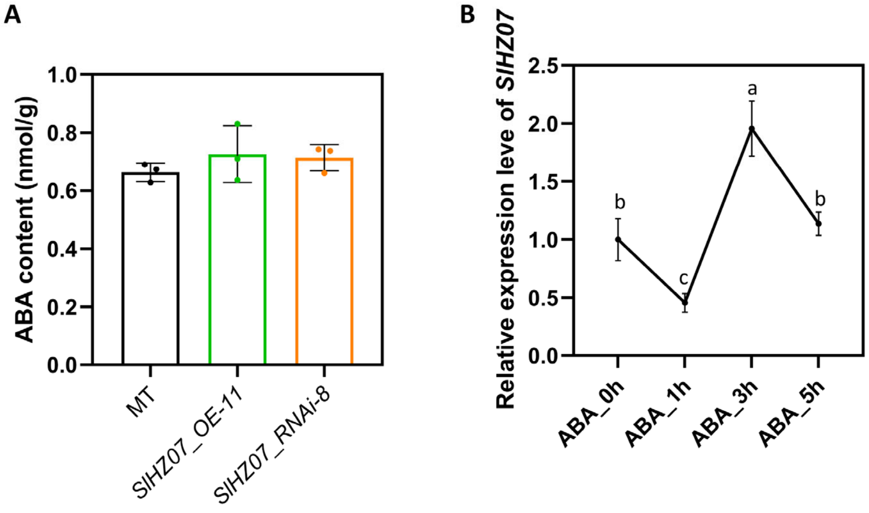

Figure S3: Relationship between *SlHZ07* expression and ABA. (A) Endogenous ABA content in leaves of MT, *SlHZ07_OE-11* and *SlHZ07_RNAi-8* at six weeks after germination. (B) Relative expression level of *SlHZ07* in detached 4th leaves of one-month-old MT plants following treatment with 100 μM ABA for 0, 1, 3, and 5 h. Data are presented as the mean ± SD (n = 3 biological replicates). Different letters indicate significant differences among treatments or genotypes ($p < 0.05$; one-way ANOVA)

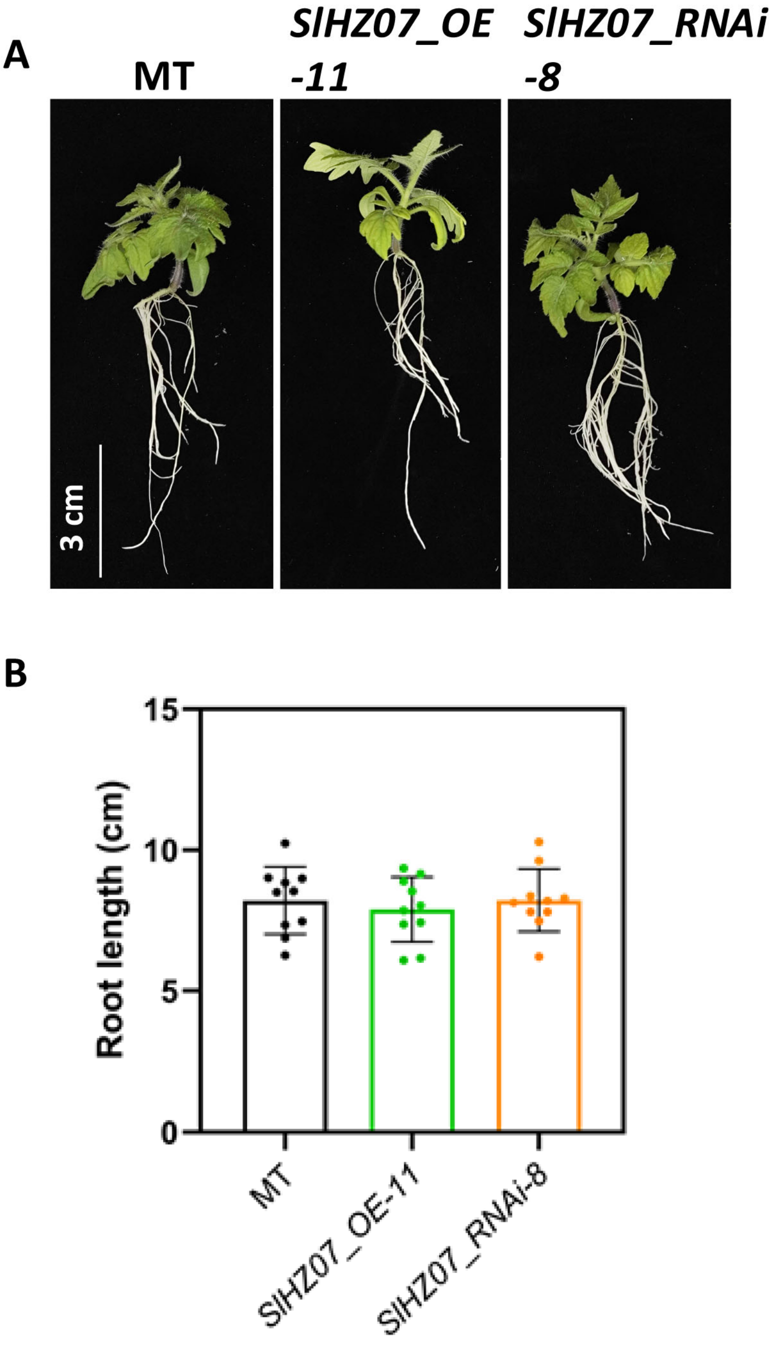

Figure S4: Root growth of MT, *SlHZ07_OE-11*, and *SlHZ07_RNAi-8* seedlings under control conditions. (A) Representative images of MT, *SlHZ07_OE-11*, and *SlHZ07_RNAi-8* seedlings grown on MS medium for 20 days after germination. (B) Primary root length of the indicated genotypes. Data are presented as means ± SD (*n* = 10 biological replicates)

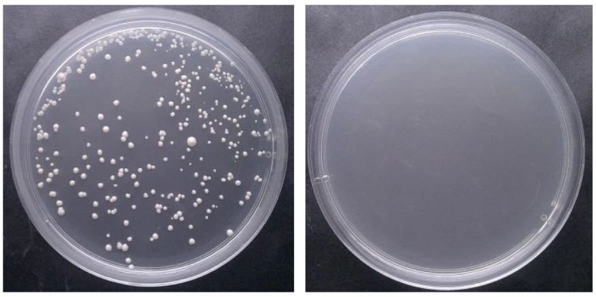

Figure S5: SlHZ07 does not bind to the *JAR1* promoter in the Y1H assay. A 550 bp *JAR1* promoter fragment containing the predicted HD-Zip II binding site was cloned into the pAbAi vector to generate the bait construct (*pAbAi-JAR1pro*). Yeast cells co-transformed with *pAbAi-JAR1pro* and *pGADT7-SlHZ07-AD* vectors failed to grow on SD/-Leu selective medium supplemented with 50 ng/mL aureobasidin A (AbA). Table S1: Primers for cloning; Table S2: qRT-PCR primers.
